# Translation of a longitudinal survey of veterans’ well-being into action by a research-practice-policy partnership

**DOI:** 10.3389/fpubh.2024.1346057

**Published:** 2024-12-24

**Authors:** Daniel F. Perkins, Kimberly J. McCarthy, Nicole R. Morgan, Brandon A. Balotti, Katie E. Davenport, Keith R. Aronson, William Lockwood, Megan Andros

**Affiliations:** ^1^Clearinghouse for Military Family Readiness at Penn State (Clearinghouse), The Pennsylvania State University, University Park, PA, United States; ^2^Department of Agricultural Economics, Sociology, and Education, The Pennsylvania State University, University Park, PA, United States; ^3^Social Science Research Institute, The Pennsylvania State University, University Park, PA, United States; ^4^Department of Biobehavioral Health, The Pennsylvania State University, University Park, PA, United States; ^5^May & Stanley Smith Charitable Trust, Corte Madera, CA, United States; ^6^The Heinz Endowments, Pittsburgh, PA, United States

**Keywords:** veterans, partnerships, coalitions, applied research, military-to-civilian transition, scholarship, policy, longitudinal survey

## Abstract

**Introduction:**

Research-practice-policy partnerships are shifting the academic research paradigm toward collaboration and research-informed action at community and policy levels. In this case study, researchers partnered with philanthropic foundations to actualize data findings from a rigorous, longitudinal study.

**Context:**

In 2016, a survey of post-9/11 military veterans began assessing veterans’ well-being in key domains: health, vocation (education and employment), finances, and social relationships. Data were collected from 9,566 veterans with three study aims: document factors affecting well-being, describe the use of transition-assistance programs and distill them into common components, and identify components associated with positive changes in veterans’ well-being.

**Partnership formation and priorities:**

The study evolved into a partnership among an academic applied research center and philanthropic funders to disseminate survey findings, investigate additional research questions of practical application, and help ensure public and private funds are invested in evidence-informed programs and services that support veterans’ well-being. Four RPP partnership goals were identified.

**Mechanisms and actions:**

Goal 1 included survey expansion to capture data on emerging concerns (e.g., COVID-19 impacts, educational experiences, burn pit exposure, civic engagement, social-media use). This resulted in eight waves of data collection over 6.5 years. Goal 2 involved co-interpretation of data to define successful military-to-civilian transition (MCT) and strategic communications to engage national leaders in policy change for veterans. Goal 3 focused on evaluation support of partners’ organizational portfolios and programs, which resulted in new tools such as an online screener that veteran-serving providers could use to identify veterans’ MCT risks and respond with tailored, research-informed resources and program components. Goal 4 allowed for the application of research findings with an innovative model for using longitudinal study variables within robust comparison analyses to assess partners’ program components; propensity matching demonstrated that effective component use leads to better outcomes for veterans (e.g., higher salaries).

**Discussion:**

Partnerships can equip funders and service organizations with credible data, clear messaging, and tools to advocate for and champion the well-being of populations. This partnership, galvanized by using data for co-learning and collaborative action, has augmented the nation’s understanding of veterans’ reintegration and has led to veterans receiving data-driven support for successful transitions and enhanced well-being.

## 1 Introduction

A shift has occurred in the paradigm that places research innovation exclusively within universities. Some research efforts now reflect relationship building and inclusion of partners’ discernments rather than sole reliance on researchers’ expertise. Cooperative research models and frameworks involve the individuals and communities that will be affected by research, such as collaborative community-engaged research and participatory action research (1–4). In addition, the natural evolution of research-practice-policy (RPP) partnerships, primarily in the educational sector, has led to a greater understanding of links between aspects of partnership effectiveness and improved outcomes and equity in practice and policy: long-term collaborations, diversity among partners, a participatory and practical research approach, and capitalization of partners’ multifarious expertise (5).

Traditionally, research has been recognized as cooperative but not necessarily collaborative—research was conducted for a research audience and failed to engage partners in identifying needs, framing issues, or utilizing data findings to glean new perspectives regarding problems and solutions (5). However, RPPs have been hypothesized as having the potential to thwart a “historical and persistent imbalance of power: the researcher’s long-exercised power to define the focus of research without giving participants a say in purposes and methods, in effect, turning them into subjects who lack voice or power” (5) (p. 10). Impactful RPP partnerships involve researchers and partners (e.g., educators, practitioners, funders) in a shared power dynamic that supports leveraging assets.

**Figure 1:**
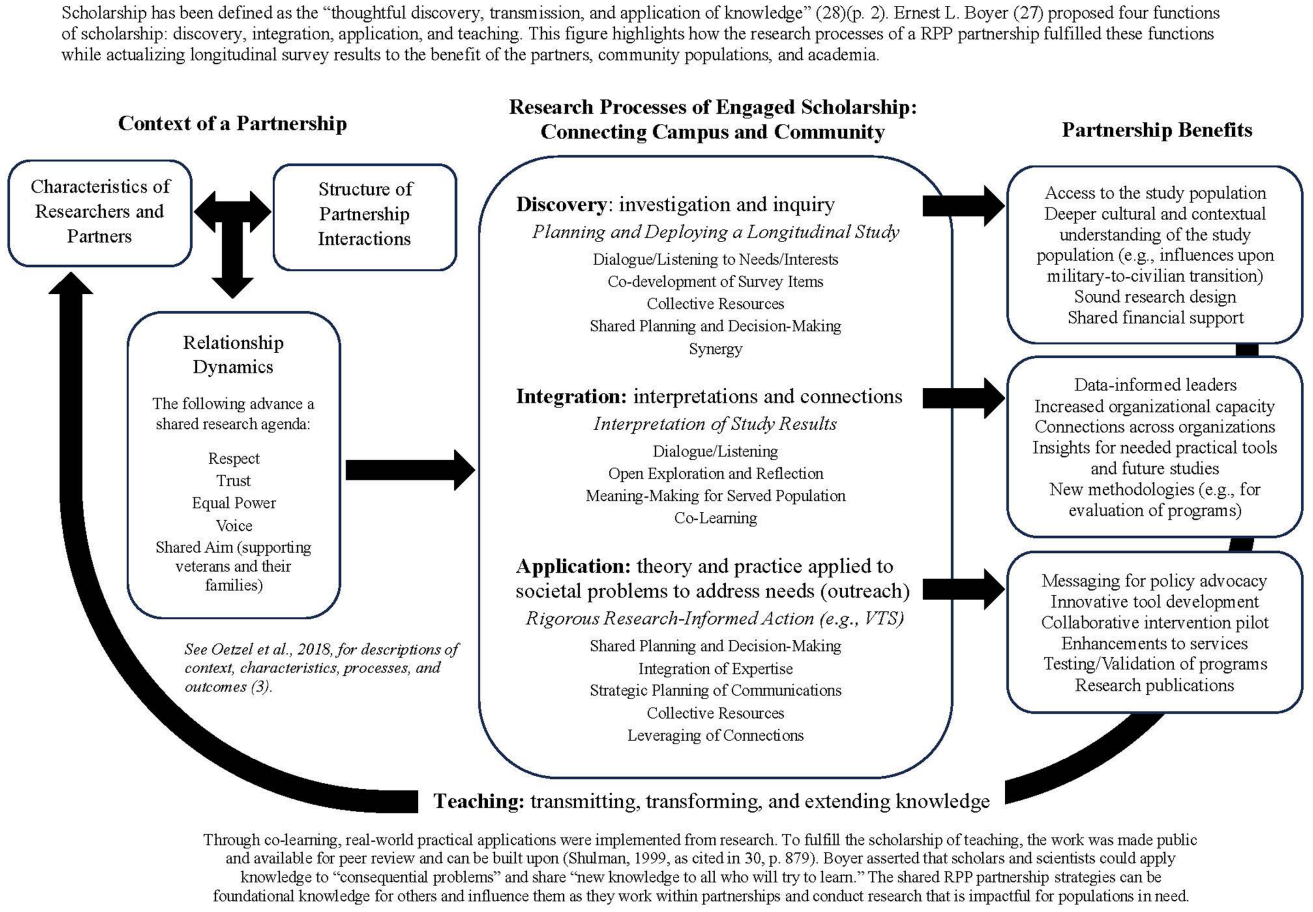
The power and potential of partnerships: actualizing the functions of scholarship. Scholarship has been defined as the “thoughtful discovery, transmission, and application of knowledge” (28) (p. 2). Ernest L. Boyer (27) proposed four functions of scholarship: discovery, integration, application, and teaching. This figure highlights how the research processes of a RPP partnership fulfilled these functions while actualizing longitudinal survey results to the benefit of the partners, community populations, and academia.

Collaborative research design, decision-making, and delivery can drive innovative programs, practices, and policies. Our research team partners with philanthropic foundations and has planned, conducted, and actualized data findings from a large, longitudinal study of post-9/11 veterans. The RPP partnership shares the aim of community-centered models: to advance practice within local contexts. The partnership’s collaborative premise is a collective conceptualization of improvements to existing practices and service delivery; researchers’ involvement emphasizes joint planning and expansion of data collection and analysis while partners inform interpretation and implications of data findings (2, 6–8). As a result of the partnership, research, practice, and policy have been advanced. For example, a novel program evaluation methodology was created, legislative action was influenced, and a risk-screening tool was developed for veteran-serving providers. The RPP partnership’s effectiveness has depended on the dimensions put forth by Henrick et al. (9), which include cultivating trusting relationships, conducting rigorous research to inform action, supporting partners in achieving goals, producing knowledge for improvement efforts, and building partners’ capacity to engage in work. Fulfilling each dimension has been a step toward the application of science to real-world solutions. Societal problems are complex, so scholarship must be utilitarian and broadened to better address the needs of global populations. This can be better achieved if teaching, research, and service are valued equally within academia (see Figure 1).

## 2 Context: longitudinal survey was the impetus for partnering

Joint participation in a longitudinal study sparked interest in the formation of the RPP partnership and later furthered research and engendered services and tools to the benefit of post-9/11 military veterans. The longitudinal study, which launched in 2016, was The Veterans Metrics Initiative: Linking Program Components to Post-Military Well-Being Study (TVMI). This research effort was coordinated by the Henry M. Jackson Foundation for the Advancement of Military Medicine, Inc. (HJF). TVMI leveraged the strengths of federal, private, and tier-one research universities. Six Co-Principal Investigators designed the study protocol with input from a scientific advisory board and various stakeholders and were responsible for survey development, survey deployment, and data analysis. The researchers represented the United States Department of Defense (DoD), the United States Department of Veterans Affairs (VA), academia through the Clearinghouse for Military Family Readiness at Penn State (Clearinghouse), and private industry (ICF International, Inc.). HJF also assembled multiple public and private philanthropic organizations to financially support the study.

**Figure 2:**
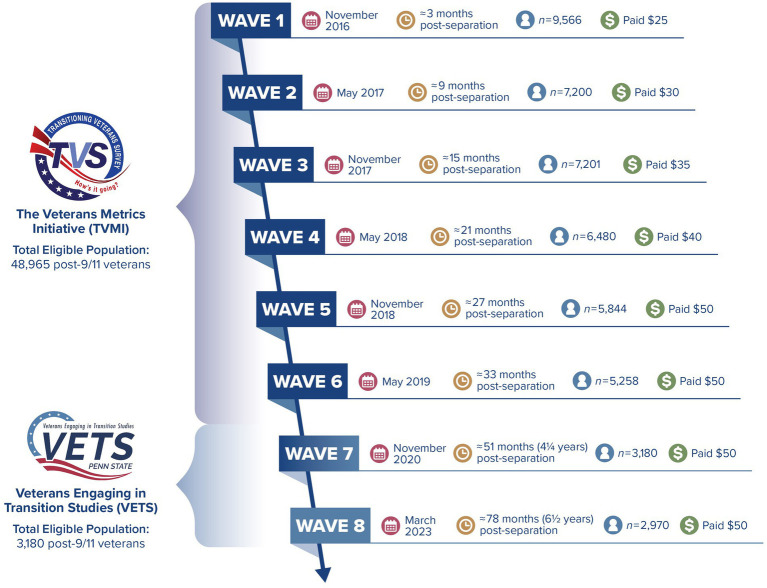
Timeline of longitudinal veteran study.

TVMI was the first longitudinal investigation that specifically examined military-to-civilian transition (MCT) with a national sample of post-9/11 United States (U.S.) veterans. The study included the following goals: (1) document veteran well-being during the MCT and categorize factors predicting changes in well-being in four domains [i.e., finances, mental and physical health, vocation (i.e., employment, education), and social relationships], (2) identify programs that veterans use during civilian reintegration and distill them into their common components, and (3) examine the links between common components and veteran well-being to identify effective program components. Study eligibility criteria included (a) at least 180 days of military service as an officer, warrant officer, or enlisted personnel who had separated from one of four active duty component Service branches (i.e., Army, Navy, Air Force, Marine Corps) or (b) deactivation from active duty status after serving at least 180 days in a reserve component (i.e., Army National Guard, Air National Guard, Army Reserve, Air Force Reserve, Navy Reserve, Marine Corps Reserve). A total population of 48,965 veterans were identified as separated or deactivated from active duty status in the 90 days prior to the date of contact information extraction from the VA/DoD Identity Repository. There were six waves of TVMI data collection with surveys administered at approximately 6-month intervals between November 2016 and May 2019. For further information [see Figure 2 and Vogt et al. (10)].

The RPP partnership did not coalesce until after TVMI data collection concluded in 2019. During TVMI survey development and deployment, researchers and funders met every 6 months. Although reliable measures of practical importance were co-created, the philanthropic organizations had invested in survey administration to use data findings in their programming and grant-making efforts to the benefit of post-9/11 veterans, and after survey data were initially analyzed, these funders were left desiring further co-interpretation of the data findings and the collaborative design of tools and services to meet identified veteran needs. To rectify shared disappointment in the usability of the data set thus far, one of the TVMI Co-PIs proposed a research hub for post-9/11 veteran studies to further TVMI data interpretation in a partnering effort that was distinct from the original TVMI study team.

## 3 Partnership formation and priorities

In 2020, without any funding, the hub, which became known as the Penn State VETeran Evaluation and Research Applications Network (Penn State VETERANetwork), was created by the Clearinghouse. This RPP partnership addresses questions and issues, as identified by foundations and non-profit organizations, related to post-9/11 veterans’ transitions to civilian life. The hub’s primary objective is to help ensure public and private funds are invested in evidence-informed programs and services that support veterans’ and their families’ well-being. The hub has engaged 19 foundations in data-collection and utilization efforts. Interactions are also facilitated with other organizations when strategic opportunities arise (e.g., Institute for Veterans and Military Families at Syracuse University and University of Southern California’s Military and Veterans Programs).

At the partnership’s inception, the following goals were identified by Clearinghouse researchers for the partnership’s initial strategic direction; each goal was informed by partner needs and focused on establishing a joint research agenda and shared data translation among the partners. Actualization of these goals illustrates how research uptake resulted from the partners jointly interpreting and using survey data for research-informed action.

1. Continue to survey a large subsample of post-9/11 veterans from TVMI to address partner-initiated questions about the transition experiences of post-9/11 veterans.
2. Engage in data analyses to inform strategic communication efforts that contribute to the national dialogue and policies regarding post-9/11 veterans.
3. Offer scientific and evaluation consultation to partners for their veteran grant-making portfolios.
4. Conduct applied research, including evaluation of partners’ programs for post-9/11 veterans during their MCT.

### 3.1 Data drives partnership priorities

When the Penn State VETERANetwork was formed, the research team listened to the partners to drive further data analysis, new data collection, and action. The partners were in positions of influence and could interpret the TVMI findings for systems operating within the veteran space. The first task of the hub was to prioritize the research topics and research questions the partners suggested for discussion and decision-making. Each partner proposed research questions, and these were categorized into eight themes for a survey: employment, entrepreneurship, financial well-being, health and wellness, learning/education/training, metrics, MCT, and veteran vs. civilian comparisons. The highest collective priorities emerged as veteran employment and understanding aspects of a successful MCT. In addition, partners ranked thirty proposed research questions, and those named as a first or second priority by any partner were deemed high-priority. These included characteristics of successful and unsuccessful transitions, veterans’ and veteran spouses’ pursuit of entrepreneurial goals, and metrics by domain for each of the foundations to track program progress and effectiveness. Lastly, partners ranked topics within each of the eight categories and the collective means were used to determine priorities within the categories. For example, within the employment category, the partners ranked 14 items and were most interested in demographic trends for employability and an analysis of females and workforce outcomes.

## 4 Mechanisms and actions of the partnership

The intention behind developing the Penn State VETERANetwork was continued collaboration, and as partners further explored the research questions designed for and studied in TVMI, more inquisitive and mature reflection emerged and guided collective discovery. Shared data translation drove decision-making around further data analysis, policy efforts, and program enhancement.

Although no quantitative data has been collected on the partnership’s dynamics, a variety of indicators strongly suggest the effort is working and will lead to positive outcomes. Interactions have been marked by characteristics reported in prior research: trust, humility, commitment, motivation, mutualism, resourcefulness, reciprocity, and reflexivity (5, 11). Power sharing has been a key mechanism that reflects humility, trust, and reciprocity. The Clearinghouse researchers adopted a service leadership philosophy with a focus on the vitality of the partnership and promoting the use of data to foster the well-being of veterans. While the research team assumes a coordination role to ensure regular communication and facilitate ongoing data investigation sessions, neither the researchers nor any of the partners assume an authoritative or decision-making role. This coupled with transparency, led to improvements in the partners’ approaches to external communications and services. Mutualism and reflexivity are embodied in the group’s reflection, which has been a key mechanism for fostering the team ethos that has led to the successful use of data. The partners are willing to remain flexible, listen, dialog, and “follow the breadcrumbs” of sensemaking as data have been combined with partners’ knowledge of and self-experiences as veterans. Sample outputs demonstrate the partners’ commitment, motivation, and resourcefulness including consistent participation in a monthly webinar series since 2020, which reflects continued dedication to data interpretation and utilization; the partnerships’ ability to institutionally and financially sustain engagement through the procurement of 8 grants totaling over $2.2 million; expansion of the TVMI longitudinal survey through two additional waves of data collection with approximately 35% of the content reflecting new survey questions proposed by the partners; acceptance of 15 journal publications communicating the collaborative interpretation of survey data results; and eight federal policy interactions (e.g., meetings with congressional staffers, informational presentations to Senate and House Veterans’ Affairs Committees). Further examples of partnership processes and intended outcomes are highlighted below within each of the partnership’s goals.

### 4.1 Continuing survey efforts

Partners were invited to inform and be informed by the TVMI data analyses. TVMI initially collected six waves of data across 3 years, and it provided the partners a greater understanding of the risks veterans face. However, RPP partnerships are not limited in scope; they evolve and are enduring. In 2020, the partners’ interest in continued data collection and interpretation resulted in an expansion of TVMI.

The partners moved beyond the initial goals of TVMI data collection and extended the longitudinal survey through a new survey called the Veterans Engaging in Transition Studies (VETS; see Figure 2). During the co-production of this survey, the partners were queried about emerging data interests and changing circumstances in the veteran space, and new lines of questions were added to the original TVMI survey during survey design (e.g., higher education experiences and debt obligations, impacts of the COVID-19 pandemic). The VETS survey launched in early winter 2020. The TVMI sample had been contacted, and a large subset of the sample agreed to participate in the expanded VETS survey effort (Wave 7, *n =* 3,180, 51 months since separation). An 8th wave of longitudinal data collection occurred in 2023, which was 6 ½ years post-separation for the survey participants (*n =* 2,970). VETS development and deployment funders included a sub-set of the hub partners: Arthur M. Blank Family Foundation, the May and Stanley Smith Charitable Trust, The Heinz Endowments, The Pew Charitable Trusts, and the Wounded Warrior Project, Inc.

Steps were taken early in the partnership’s development to ensure usable survey results. A monthly webinar series of data-investigation sessions was initiated and continues to be held; the research team shares survey data analyses followed by data-translation discussions. During each webinar, a new topic, based on the partners’ requested data analyses, is explored, such as disparities in risk for specific veteran subgroups (e.g., junior enlisted paygrades and unemployment and persistent problematic financial status, females and military sexual trauma (MST), veterans with poor social connections/low social satisfaction and mental health concerns). In addition, a website with a password-protected partners’ portal was established to share materials, such as webinar recordings, data reports, and publications. Furthermore, in 2021, independent feedback sessions were conducted between the research team and each foundation to better understand each organization’s role in supporting veteran well-being and to make the data actionable for each organization’s efforts. This process of engagement with implementing partners and users was imperative to continuous quality improvement, and it aligned with the AIDED (Assess, Innovate, Develop, Engage, and Devolve) model’s steps of assessing the environmental context and innovating to fit user receptivity (12). The result was a list of products, tools, services, and future analyses that could be used to guide the strategic direction of the RPP partnership.

**Figure 3:**
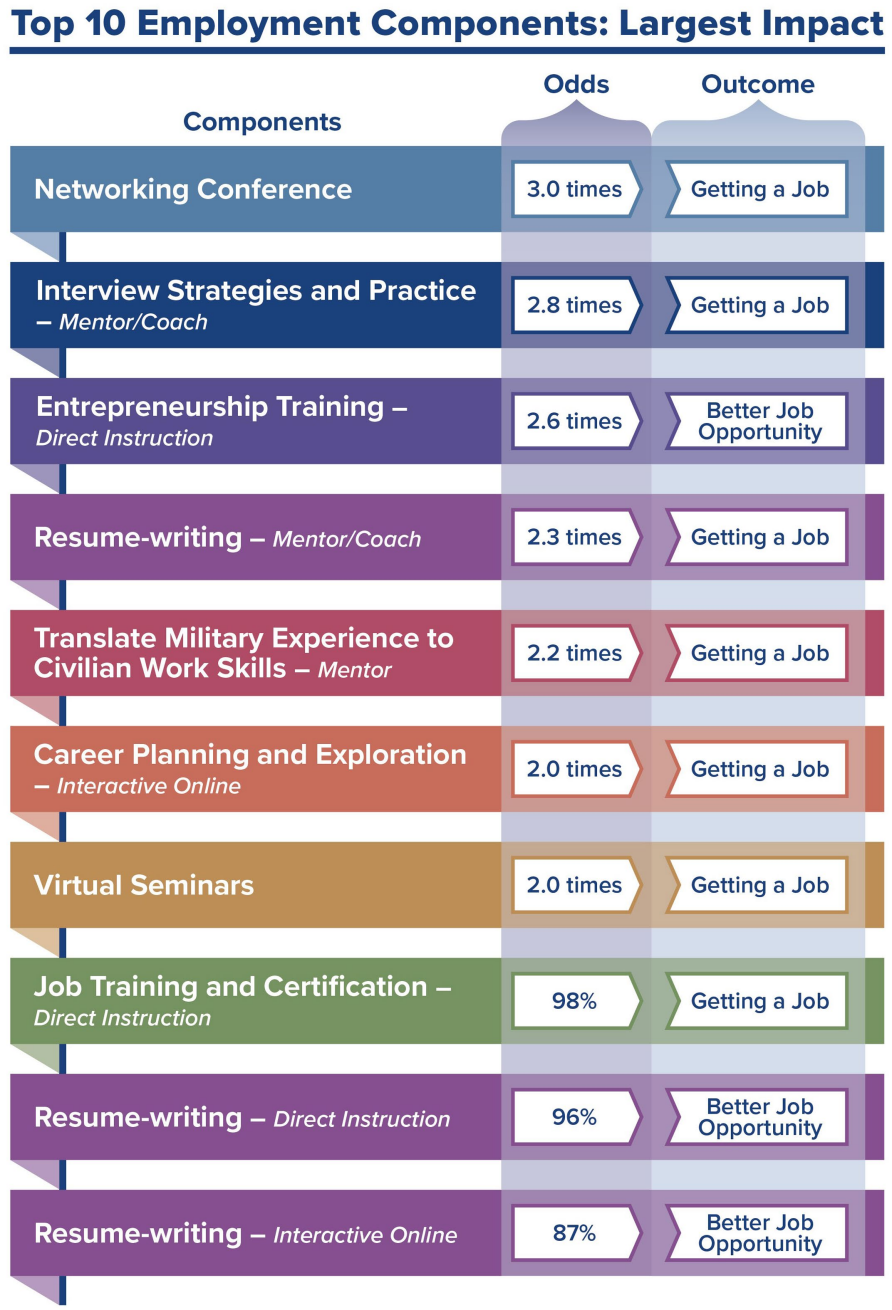
Adapted common components analysis: employment example.

A primary focus from the hub’s inception has also been to use data to define successful MCT by exploring risks and protective factors. During TVMI, an adapted Common Components Analysis (CCA) was developed to identify components of the programs and services used by the surveyed veterans in the four well-being domains (13). Traditionally, a CCA distills the results of program evaluations from randomized control trials into key elements that produce intended outcomes. In the adapted CCA, program components were studied in four primary areas (i.e., content, process, barrier reduction, and sustainability), and common components were tested to identify associations with changes in intended program outcomes (e.g., employment, job retention). As the partners’ original interest in the research was to determine how best to strategically fund prevention and intervention programming for veterans, the adapted CCA findings have contributed to their uptake of data findings for programmatic funding and improvement (see Figure 3 for an employment example).

### 4.2 Goal 2: strategically informing policy

RPP partnerships are impactful when they shift the research paradigm from academic data collection to collaboration that uses evidence to inform action. The partners decided the TVMI and VETS survey data findings needed to be actionable, so scientific evidence could be applied to policy discussions. The partners agreed that engaging in strategic communications could support governmental leaders’ capacity to understand and, consequently, incorporate data findings into improving practices and policies for veterans. Data were used to define successful transition and equip multiple initiatives related to topics such as suicide prevention, economic opportunities, employment and underemployment, and transition needs. One of the policy-focused efforts is highlighted next.

Partners, who were representatives of foundations with established legislative relationships, served as liaisons between legislative staffers and the RPP partnership. They leveraged their connections to share research findings related to MST, and, in real-time, the research team responded to Senators’ and staffers’ questions with in-depth analyses regarding MST. The data set had been analyzed across many outcomes, and the rate of MST reported by the surveyed veterans exceeded the rate reported by the VA. In TVMI, more females than males reported unwanted sexual attention (41% vs. 3.2%) and unwanted sexual contact (17.4% vs. 1.1%) during their military service. The policy implications were discussed, and the data were used to advance the *Military Justice Improvement and Increasing Prevention Act*. This Act contributed to a defense bill that moved investigation and prosecution from the military chain of command to an Office of Special Trial Counsel and enacted sexual-assault prevention provisions, such as better training (see Table 1). Thus, research uptake can be facilitated when partners’ social connections and expertise are coupled.

**Table 1:** Comparison of rates for military sexual trauma as reported by service members and veterans.

	Annual Report on Sexual Assault in the Military for FY 2016^a^ (Service member estimates from *The Workplace and Gender Relations Survey for Active Duty Members*)	United States Department of Veterans Affairs’ Provider Screening Rates^b^ (veterans receiving United States Department of Veterans Affairs healthcare)	The Veterans Metrics Initiative (representative sample of post-9/11 veterans surveyed from 2016-2019) *n =* 7,741 for females *n =* 40,743 for males
Military women	4.3% experienced sexual assault in 2015	1 in 3 reported experiencing military sexual assault	41% reported unwanted sexual attention, and 17.5% reported unwanted sexual contact during military service
Military males	6% experienced sexual assault in 2015	1 in 50 reported experiencing military sexual assault	3.2% reported unwanted sexual attention, and 1.1% reported unwanted sexual contact during military service

Rates reported by the post-9/11 veterans in The Veterans Metrics Initiative study were higher than those reported by the United States Department of Veterans Affairs but consistent with other published research. The United States Department of Defense’s 2016 annual report suggested that 14,900 Service members experienced some form of sexual assault in 2016; this rate was reduced from 20,300 Service members in 2014. For more information on the United States Department of Defense’s Sexual Assault and Prevention Response Office’s initiatives and current rates, access https://www.sapr.mil/.

a United States Department of Defense. Department of Defense Annual Report on Sexual Assault in the Military – Fiscal Year 2016 [Internet]. Washington D.C.: Sexual Assault Prevention and Response Program; 2016. [cited 2023 Sept 26]. Available from: https://www.sapr.mil/public/docs/reports/FY17_Annual/FY16_Annual_Report_on_Sexual_Assault_in_the_Military_Full_Report3_Volume1.pdf.

b Fact sheet: Military sexual trauma [Internet]. Washington D.C.: United States Department of Veterans Affairs; 2021 May. [about 3 screens]. Available from: https://www.mentalhealth.va.gov/docs/mst_general_factsheet.pdf.

Some funders, such as the William T. Grant Foundation (Note: They are not a member of the Penn State VETERANetwork), support studies that examine whether and how RPPs increase the use of research in policymaking (14). Academic institutions can serve as a resource to policy leaders and practice stakeholders as they fund and deliver programs and services. Strong examples of evidence dissemination for social-investment optimization exist, such as Evidence-based Prevention and Intervention Support (known as EPIS) at Pennsylvania State University. Collaborative use of evidence by higher education, state and federal agencies, and private industry and funders is a growing governmental priority. The Biden-Harris Administration’s “Year of Evidence for Action” and the first-ever White House Summit on Evidence for Action, which was co-hosted by the Office of Science and Technology Policy and the Office of Management and Budget in early 2023, is an example of this (15).

An underlying mechanism for influencing policy is recognizing that policymakers cannot and do not need to be experts in topics to produce sound policy. Our RPP partnership shared the applied science behind existing societal problems. This promoted nonpartisan, critical thinking of core issues and resulted in solutions research has shown to be effective and efficient.

### 4.3 Goal 3: supporting partners’ portfolios with scientific consultation

Partners have verbalized that engagement has been beneficial, and they have sought to expand on the original research agenda to identify additional practical data applications. Each foundation intends to meet its own organizational and philanthropic objectives. Similarly, the research team has been accountable to its peers through journal publications and presentations that communicate data findings to the research community; however, those academic efforts are considered secondary to actively collaborating with the partners. The primary focus has been translating data into meaningful action for the partners’ current and future grant portfolios and to advance the nation’s commitment to veterans. This work ranged from realigning portfolio grantees’ logic models with intended outcomes to creating visualizations of veteran, program, and geographic data to tell the story of post-9/11 veterans’ experiences and outcomes. An example of elevating data findings to the next level of usability follows.

A key takeaway from the TVMI study was that a screener that addressed veteran risk factors and program-component needs (e.g., interviewing practices for those looking for a job, social programs for those dealing with feelings of isolation) could enhance veteran-serving providers’ effectiveness (e.g., from prevention to treatment programs). Thus, the Veteran Transition Screener (VTS) was developed to help providers determine the level of support needed by the veterans with whom they work and to match veterans’ needs to the type of program components that are most likely to result in positive well-being outcomes. The online screener contains seven sections: background information (i.e., demographics), specific experiences (i.e., combat experiences, moral injury, adverse childhood experiences, MST, and VA disability rating), and a section for each of the well-being domains. Given the focus differences of veteran-serving organizations, providers can select which sections they give to their clients; the background information and specific experiences sections are completed by all clients as the categories in these two sections were found to be robust predictors of risk factors for MCT success across all well-being domains (see Table 2). In 2022–23, a pilot of the VTS was funded by one of the hub’s partners and was conducted in the field with veteran-serving providers to identify areas for improvement (e.g., items, structure, and reporting). The pilot providers included a cross-sector collaborative, based in San Diego, California, who matches transitioning Service members with Peer Navigators to address challenges related to employment, housing, injury, and MCT, and a national non-profit that provides employment support to Service members, veterans, and military spouses. The VTS was refined following strategies outlined in the Plan-Do-Study-Act Cycle and Model for Improvement (16, 17) based on feedback from the veteran-serving providers and collected client data. Currently, the VTS is being built into a learning-management system to automatically generate reports. VTS, and other applications of data, are positively impacting service providers’ ability to meet veterans’ needs using evidence-informed strategies.

**Table 2:** Veteran Transition Screener: veteran risks and provider recommendations.

Well-being domains	Identified risk factor(s) for veterans	Examples: programs and common components recommended to service providers
Vocation (Employment only)	Paygrades: E1 to E4 Discharge status: medical Gender: female Race/ethnicity: all minorities United States Department of Veterans Affairs’ disability rating: 10% to 100%	Interviewing skills through a mentor/coach Resume writing by reading online or through online tools Career planning with direct instruction, a mentor/coach, or a networking group Attendance at virtual career fairs
Financial	Paygrades: E1 to E4 Discharge status: medical Gender: female Race/ethnicity: Black; Hispanic United States Department of Veterans Affairs’ disability rating: 10% to 100%	Assistance with financial planning and protection Assistance with accessing United States Department of Veterans Affairs’ benefits Accessing credit score
Physical health	Paygrades: E5 to E6	Primary care physical referral VA clinic referral Preventative/diagnostic/treatment health services Wellness programs
Mental health	Paygrades: E1 to E3; E5 to E6 Adverse childhood experiences Combat exposure Moral Injury	Mental health counseling Crisis hotline information Safety planning tools (e.g., Suicide Safety Planning Tool, Brief Suicide Safety Planning Tool) Wellness programs (e.g., weight loss, de-stress, health advocacy)
Social support	Paygrades: E6 Moral Injury Traumatic Brain Injury symptoms	Social activities and organizations Mental health counseling

Risk factors were identified for post-9/11 veterans based on significant associations with detriments in the respective well-being domains during analysis of The Veterans Metrics Initiative longitudinal data set, and these risk factors are shown in column two for each of the four assessed well-being domains. An adapted Common Components Analysis identified effective programs and common components for use in countering risks (13). When the Veteran Transition Screener is administered to veterans, service providers are shown programs and common components to recommend in response to the veterans’ self-reported risks during tool use (see column three). If veterans use the recommended programs/common components, improvements in outcomes in the targeted well-being domain(s) may result.

### 4.4 Goal 4: conducting applied research and program evaluation

Foundations often invest in programs/services that vow a positive societal impact. For some populations, such as post-9/11 veterans, evidence-based programs/services are not available or do not meet all needs. Often programs/services are offered with promises, such as increased employment opportunities, with little or no evidence to substantiate these assurances. For non-profits, research effectiveness and efficacy trials are costly and time-prohibitive, and they demand a high level of research expertise. To counter limitations, the partnership strategized regarding how to utilize the CCA findings and the longitudinal data to create a ready-made comparison sample through propensity score matching. This novel approach capitalized on TVMI and allowed it to be applied within a rigorous quasi-experimental study, which was financially supported by a partnership member.

In 2021, a third-party impact evaluation of Onward to Opportunity (O2O), a career training program offered by the Institute for Veteran and Military Families at Syracuse University for transitioning Service members, veterans, and their spouses, was commissioned. O2O utilizes industry-validated curricula and career-coaching services. The research team created a valid comparison group using the TVMI dataset and program components for a comparison study. Elements of O2O programming (e.g., job training, licensing assistance) were mapped to TVMI content and process components, barrier-reduction components, and sustainability components (18). Then, O2O program-participant data were aligned to the time frame data of the TVMI sample (i.e., 2017–2019) and matched on multiple demographic characteristics, including age, gender, race/ethnicity, pay grade, Service branch, and level of education, using propensity matching.

**Figure 4:**
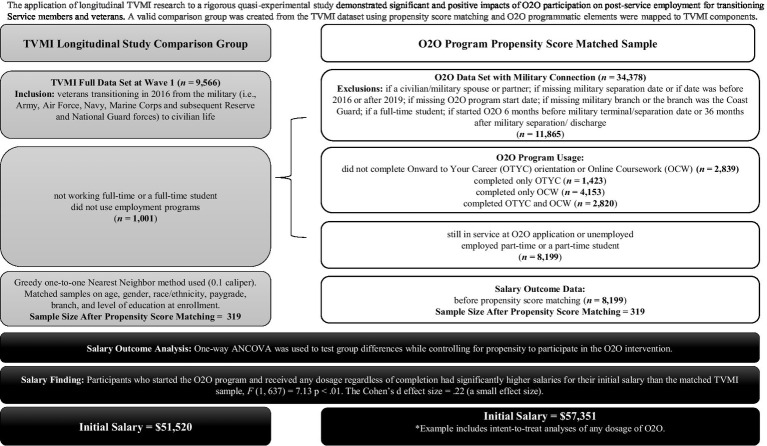
The Veterans Metrics Initiative (TVMI) longitudinal study comparison group: Onward to Opportunity (O2O) sample selection and matching process using the outcome of salary as an example. Longitudinal TVMI research was applied to a rigorous quasi-experimental study and demonstrated significant and positive impacts of O2O participation on post-service employment for transitioning service members and veterans. For the O2O outcome analyses, the methods varied based on the research question. For salary, a one-way ANCOVA was run to test group differences while controlling for propensity to participate in the O2O intervention. Logistic regression was also used to analyze leaving for a better opportunity at 6-month and 12-month follow-ups. Each outcome analysis had its own matched one-to-one sample; therefore, the sample size for the matched sample is significantly smaller than the size of the sampling frame. Power analysis was conducted to ensure an adequate sample size to detect a difference at 80% power (.05 alpha).

Next, O2O program outcomes were evaluated with two employment outcomes: starting salary and job retention (19). The analyses demonstrated significant and positive impacts of O2O participation on post-service employment for transitioning Service members and veterans (see Figure 4). For example, findings demonstrated that O2O participants obtained higher salaries, even when O2O participants did not complete their learning pathway or certification exam, than those who did not participate in employment components. This benefit was strongest for veterans who separated, discharged, or retired from military service at an E6 and lower paygrade; this subpopulation often struggles post-service, so findings that indicated their salaries averaged $13,000 higher than those who did not use O2O were promising. The analyses also showed that O2O participants who left their jobs at or before the 6-month mark were twice as likely than the comparison group to leave a job for a better opportunity. This result may reflect O2O’s foci on helping veterans identify and pursue career goals and increase skills to recognize and capitalize on career advancement prospects.

To the authors’ knowledge, O2O is currently the only veteran career-training program that has demonstrated third-party validated effectiveness for program participants with the rigor of a robust experimental design. The RPP partnership not only led to this program validation but to specific program-improvement recommendations (e.g., standardizing O2O orientation and recruiting junior enlisted Service members). Thus, the partnership’s innovative model for using longitudinal study variables within robust comparison analyses has contributed to the research field and community-level programs and services.

## 5 Discussion

### 5.1 Partnership alignment with model and framework elements

This case study highlighted that the research produced in RPP partnerships is valuable and practical. Through a collaborative process, applied research can influence partner organizations’ operational objectives, programmatic investments, policies, and population-specific outcomes. Prior models (e.g., Communities That Care) have demonstrated that data collection and interpretation can galvanize partners from diverse sectors to streamline their prevention and intervention efforts by aligning intended outcomes with targeted population needs (20). This case study expounded how a robust partnership coalesced around ongoing data analyses so that data interpretations could be contextualized and transformed into practical action for prevention and intervention strategies, new policies, and practical tools that better support veterans. With a multitude of partners’ perspectives and ongoing emersion in an exploration of longitudinal data, co-generation and expansion of ideas were cultivated, and this led to action-oriented and creative possibilities.

Farrell and colleagues (21) have recently tried to build upon the Framework for Assessing Research-Practice Partnerships, which was developed by Henrick et al. (9) and later converted into a tool by The Institute for Education Sciences (22), to assess partnership functioning in the education sector. Survey scales related to trust and relationship building, joint decision-making, capacity to use research, and partners’ flexibility are being explored to identify how RPP partnerships can increase research uptake to improve student outcomes. Research uptake can potentially ameliorate public issues (e.g., poor health, drug addiction).

The authors’ RPP partnership’s effectiveness depended on the dimensions put forth by Henrick et al. (9) [*Italicized items in parentheses denote our partnership-specific additions]: building trust and cultivating partnership relationships; conducting rigorous research to inform action; supporting the partner practice organization(s) in achieving its (*their*) goals (*a primary priority for the research team over traditional research and scholarship activities*); producing knowledge that can inform educational (*veteran*) improvement efforts more broadly; and building the capacity of participating researchers, practitioners, practice organizations, and research organizations (*and funders*) to engage in (*applied science*) partnership work. Key characteristics of the partnership include humility in acknowledging partners’ expertise and equity in power and decision-making. In addition, mutuality in interactions acknowledges the partners’ desire to understand veterans’ risks and protective factors and specific, evidence-based program components, so they can be strategic in their grant-making and programmatic efforts. Furthermore, co-learning was and is the cornerstone of all of the partnership’s development and sustainability phases, and preparation and discussion of new data analyses are the linchpin. The research teams’ coordination capacity to facilitate dialogue and interpretation of the data was increased by developing a structure for regularly scheduled communications that led to continuous dialogue among the partners; there was an early recognition that data are more valuable when presented in a format that allows for discussion of meaning and practical implications.

### 5.2 Lessons learned

The authors have referred to research generically, which can be a nebulous term, especially to community-level providers and implementers who are most frequently the direct line of service to populations in need. The fields of prevention and implementation science have demonstrated that research in the form of evidence-based programs and practices is more effective at impacting community-based outcomes. This is especially true when implementers understand the importance of and adhere to delivery fidelity, that is, the degree to which a program or practice is delivered as intended by developers (23–26). When researchers educate providers and implementers on why and how to use evidence to drive decision-making (e.g., elucidating a program’s theory of change), this acquired knowledge augments providers’ and implementers’ commitment to high-quality implementation and evaluation. In addition, the opportunity for equitable power and bidirectional learning is ignited as researchers, through collaboration, can gain a greater understanding of community needs and implementation factors from the providers’ and implementers’ real-world execution of the programs or practices. Co-interpretation of community context, implementation factors, and collected participant data can occur, and this synergy and shared knowledge can generate innovative solutions and the sustainability of programs and practices that affect positive change in outcomes for populations in need.

Similarly, researchers in RPP partnerships can collaborate and coordinate with prevention and intervention intermediaries (e.g., governmental or policy leaders and foundations or funders) to promote mutual learning and action at the community level to address the needs of veterans and other at-risk populations. Indeed, the authors implore researchers to hone their communication skills to educate intermediaries about research benefits and increase awareness of research possibilities, so interest in partnership peaks. The authors strongly advocate for researchers’ engagement in partnerships to include co-development of research that can be translated into action, so researchers, with intermediaries and practitioners, jointly address real-world applications.

Ernest L. Boyer (27) proposed the following functions of scholarship: discovery, integration, application, and teaching. This case study illustrated how RPP partnerships include elements of each of these functions (see Figure 1). The discovery stage involved the co-development and deployment of the VETS Survey. Integration occurred as the partners interpreted data results through the perspective of veteran-serving organizations. Application was demonstrated as the survey results of rigorous research informed actions such as policy interactions and tool development (e.g., VTS). Teaching occurred as the research team presented data results to the partners and as data was made publicly available to be used as functional knowledge by others (e.g., CCA, MCT definition, risk factors for veterans).

### 5.3 Limitations of efforts

Limitations exist. First, partners have focused interests and a constrained capacity to fund data analyses and dissemination infrastructures. Financial support was directed to survey development and deployment, program evaluation, and tool development. The research team, as part of an academic center, could leverage resources like the skill sets and time of data analysts, managerial and administrative employees, and web designers. External funding streams for researcher-directed infrastructures could bridge research gaps across diverse sectors and for a myriad of populations at the community level. Second, time constraints restricted the amount of data analyses and methodology or tool innovations that could be addressed at one time. Third, for researchers, applied research is often viewed within universities as less rigorous than traditional research, and service or outreach is a secondary activity that is not highly prized or rewarded. Promotion- and tenure- evaluation incentivize basic research and resident education over other types of scholarship that are impactful but more difficult to assess (28, 29). Fourth, for funders, investing in research means fewer resources for programs and services. Fifth, while an alternative to research constrained by the biomedical model and clinical trials was presented, it underscores the need to develop tools to evaluate features of effective partnerships. These tools could be used as part of continuous quality improvement efforts while team dynamics evolve.

## 6 Conclusion

This case study elucidated that scholarship alone, with its emphasis on the researcher—the expert forming and imparting knowledge, is insufficient if higher education is to be fully relevant and respected for the positive societal impacts it has the potential to realize. Collaboration and co-learning through partnership are crucial for research to become actionable. The authors’ longitudinal research not only served and taught, but co-interpretation of it paradoxically elevated and equalized the authors’ role from researchers to partners in programmatic and policy decisions, which afforded greater impacts than could be achieved in an academic silo.

## Data Availability

The datasets presented in this case study were generated and analyzed for the TVMI study and can be found in an online repository (ICPSR) at https://doi.org/10.3886/ICPSR38051.v2.
